# Polidocanol monotherapy for a superficial orbital venous malformation in a horse

**DOI:** 10.1111/vop.12997

**Published:** 2022-06-01

**Authors:** Tara M. Stonex, Ashley E. Zibura, Michael Andres, Brian C. Gilger, Annie Oh

**Affiliations:** ^1^ Department of Clinical Sciences North Carolina State University College of Veterinary Medicine Raleigh North Carolina USA; ^2^ Veterinary Emergency & Referral Group New York USA; ^3^ Department of Veterinary Clinical Sciences, College of Veterinary Medicine The Ohio State University Columbus Ohio USA

**Keywords:** horse, monotherapy, orbit, polidocanol, sclerotherapy, venous malformation

## Abstract

**Objective:**

To describe the use of 1% polidocanol as the sole treatment for a superficial orbital venous malformation in a horse.

**Animal:**

A 23‐year‐old Welsh Cobb cross gelding with a distensible swelling affecting the left lower eyelid, and secondary palpebral margin abnormalities and superficial keratitis.

**Procedure:**

Color flow Doppler ultrasonography revealed non‐pulsatile blood flow within the tortuous vascular network most consistent with a superficial orbital venous malformation appearing to involve the lateral palpebral and transverse facial veins. An intravenous catheter was placed within the lateral aspect of the venous malformation, and agitated saline was slowly injected into the vessel while simultaneously ultrasound imaging the medial aspect in which the bubbles were observed coursing across, consistent with lateral to medial flow. Contrast venography confirmed a corkscrew vessel along the ventral aspect of the orbital rim. Under standing sedation, 1% polidocanol solution was administered slowly through the intravenous catheter while manual pressure was applied on the medial and lateral aspects of the venous malformation.

**Results:**

Ultrasonography performed immediately after administration of polidocanol confirmed venous stasis, and formation of a thrombus. No adverse side effects were noted. The venous malformation and associated palpebral margin abnormalities and superficial keratitis resolved at the time of re‐examination at 4 months.

**Conclusion and Clinical Relevance:**

Polidocanol as the sole treatment for a superficial orbital venous malformation in a horse was well tolerated and led to clinical resolution. Sclerosant monotherapy may be a safe treatment option for superficial orbital venous malformations.

## 1 INTRODUCTION

Orbital venous malformations are part of a spectrum of vascular malformations classified into three categories based on hemodynamic flow: “arterial flow” are those involving arteries with high blood flow, “venous flow” are those involving veins and lymphatics with low blood flow, and “no flow” are either hemodynamically isolated or involve lymphatics. Mixed forms are present as well.^1^ Orbital venous malformations are rare and have been described in a few species, including humans,^1^ horses,^2^ dogs,^3^, ^4^, ^5^, ^6^ and an iguana.^7^ Orbital venous malformations are congenital or less commonly a heritable condition.^2^, ^3^, ^4^, ^8^

Currently, there is no recommended first line of treatment for orbital venous malformations in veterinary patients. Previously published treatments include conservative management,^4^ surgical ligation,^2^ enucleation,^7^ coil embolization,^5^ and sclerotherapy in combination with coil embolization.^3^ Surgical ligation or resection is the most invasive option short of enucleation, with complications including the inability to identify ill‐defined borders of the venous malformation, necrosis or loss of function to affected tissue, and risk of hemorrhage during operation.^2^, ^8^, ^9^ Coil embolization is less invasive compared with surgical ligation but migration of coils away from the target, hemorrhage, and incomplete occlusion can occur.^3^, ^5^ Additionally, the use of coil embolization for large venous malformations is challenging as many coils are required for thrombosis, and the coils themselves can result in a permanent mass effect.^3^ With the increased risks associated with invasive procedures in addition to general anesthesia, non‐invasive procedures under sedation, such as sclerotherapy, should be explored.

Sclerotherapy is the administration of a sclerosant directly into a vessel leading to endothelial damage, inflammation, thrombus formation, and eventual regression of the vessel.^10^ It is the gold standard treatment for vascular malformation in humans and is commonly used as the sole agent to treat orbital venous malformations. Commonly, a mixture of the sclerosant and contrast agent is used. Application of manual pressure may also be performed to prevent sclerosant from entering a draining vein that may result in non‐target sclerosis.^10^, ^11^, ^12^ There are currently no reports of sclerotherapy, as monotherapy being used to treat an orbital venous malformation in any animal. The purpose of this paper is to describe the use of the sclerosant 1% polidocanol as the sole treatment for a superficial orbital venous malformation in a horse.

## 2 CASE PRESENTATION

### 2.1 History and clinical assessment

A 23‐year‐old Welsh Cob Cross gelding presented to the North Carolina State University Ophthalmology Service for evaluation of a mass affecting the left lower eyelid. This mass had been present since birth, with gradual enlargement over time and progressive distension when the head was lowered. By the time of presentation, the lesion had enlarged in size and was causing discomfort. The patient had been treated with 0.3% ofloxacin ophthalmic solution (Akorn Operating Company LLC) once daily for 1 week prior to presentation.

**FIGURE 1:**
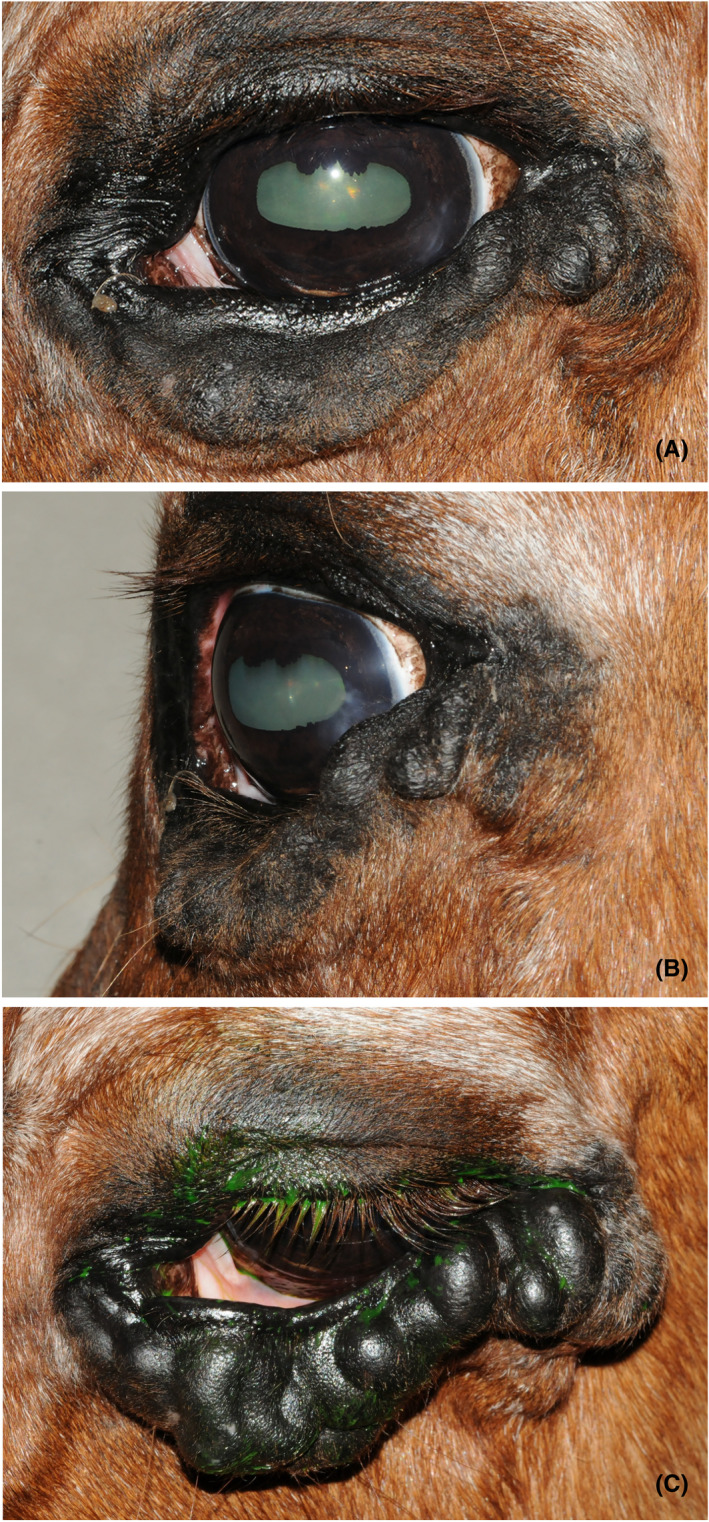
External photographs of the left eye of a 23‐year‐old Welsh Cob Cross gelding diagnosed as a superficial orbital vascular malformation of the lower eyelid. (A) Abnormalities on ophthalmic exam included a large, tortuous lower eyelid swelling, moderate ectropion of the medial lower eyelid, and (B) mass‐effect of the lateral lower eyelid resulting in ocular irritation, and focal vascularization and fibrosis of the lateral cornea. (C) The swelling distended when the head was lowered following sedation

Complete ophthalmic examination was performed by a board‐certified ophthalmologist, including slit‐lamp biomicroscopy, indirect fundoscopy, fluorescein staining, and rebound tonometry. A large, tortuous swelling affecting the left lower eyelid was identified (Figure 1A), in addition to moderate ectropion of the medial lower eyelid, and mass‐effect of the lateral lower eyelid resulting in ocular irritation, and focal vascularization and fibrosis of the lateral cornea (Figure 1B). Symmetric, bilateral retinal pigmented epithelium colobomas were also seen on fundic examination as an incidental finding. There was no fluorescein stain uptake in either eye, and intraocular pressure was 8 mmHg OD and 9 mmHg OS (Icare® Tonovet, Icare). The remainder of the ophthalmic and physical examinations were unremarkable. The patient was tentatively diagnosed with a superficial orbital vascular malformation of the lower eyelid with palpebral margin abnormalities and superficial keratitis.

### 2.2 Diagnostic imaging and treatment

**FIGURE 2:**
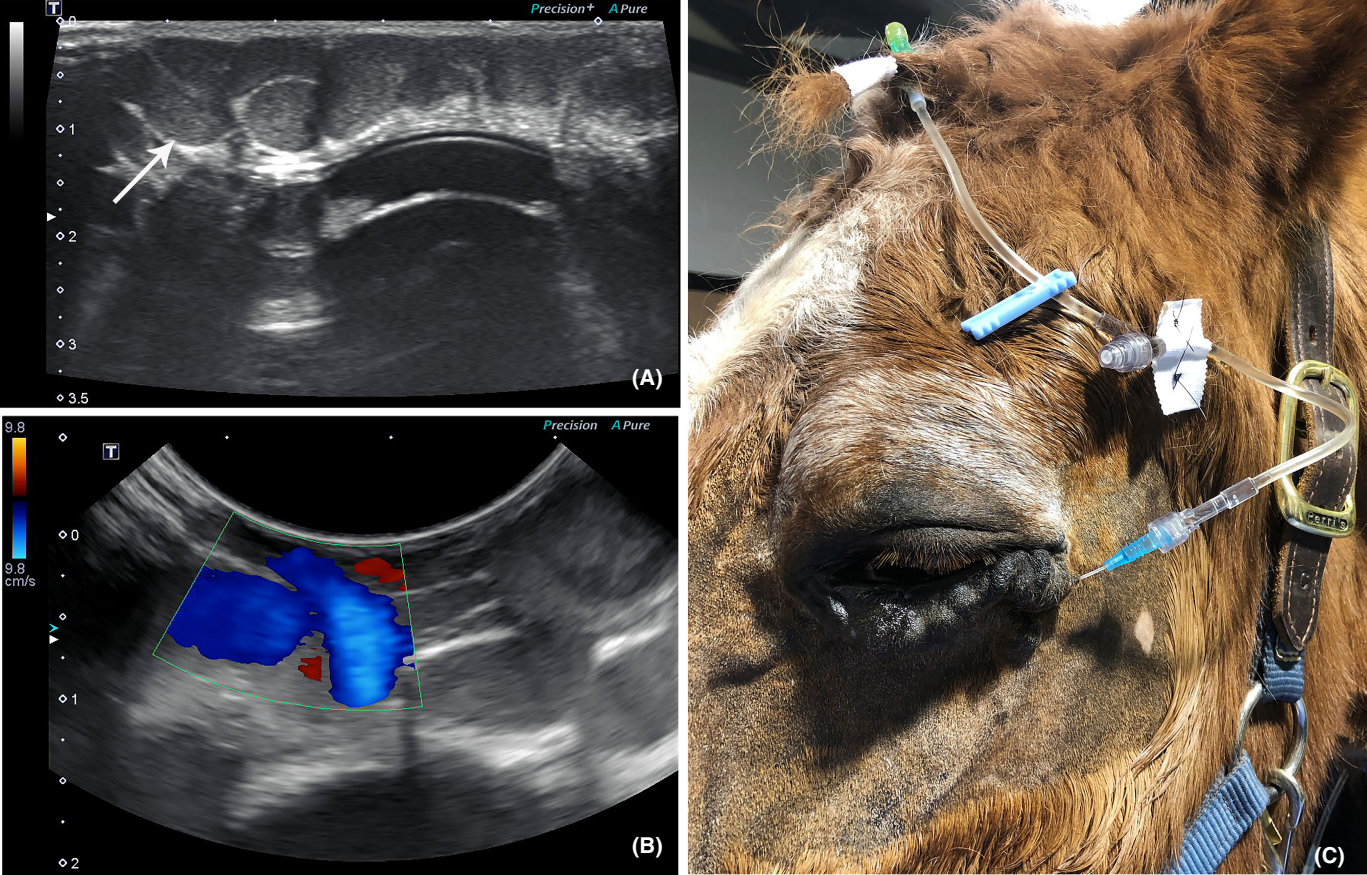
Ultrasound images of the orbital vascular malformation prior to the administration of 1% polidocanol. (A) B‐mode ultrasonography showed the presence of a large, tortuous, tubular structure superficially within the inferior palpebra (white arrow). (B) Color flow Doppler ultrasonography revealed non‐pulsatile blood flow within this structure. (C) External photograph of the superficial orbital venous malformation following placement of the 22‐gauge catheter and extension set

Ultrasonography (B‐mode and Doppler) and contrast venography were performed to confirm diagnosis and characterize flow. The patient was sedated with 400 mg xylazine (Rompun® Bayer HealthCare, LLC) intravenously (IV) and administered 500 mg flunixin meglumine (Banamine®, Merck Animal Health) IV. An auriculopalpebral and frontal nerve block were performed with 1 ml 2% lidocaine each (Sparhawk Laboratories, Inc.). The periocular area was clipped and aseptically prepared. B‐mode ultrasonography identified a large, tortuous, tubular structure superficially within the inferior palpebra measuring up to 0.45 cm in diameter and approximately 4.5 cm in length (Figure 2A). Color flow Doppler ultrasonography revealed non‐pulsatile blood flow within the tortuous vascular network, most consistent with a venous malformation appearing to involve the lateral palpebral and transverse facial veins (Figure 2B). The estimated volume was approximately 14 ml based on the length and width of the malformation.

A 22 gauge over‐the‐needle catheter was placed within the lateral aspect of the superficial orbital venous malformation using ultrasound guidance, attached to an extension set, and sutured in place using 2–0 nylon (Ethilon®, Ethicon; Figure 2C). Pulsating blood flow out of the catheter hub was not observed. Agitated saline was then slowly injected via the catheter and bubbles were noted on ultrasound coursing through the medial aspect of the venous malformation consistent with lateral to medial flow. This confirmed that placement of the catheter within the lateral aspect of the malformation was appropriate for administration of the sclerosant. An additional bubble study was performed using the same injection site while imaging the left jugular vein to confirm no immediate communication with the malformation.

**FIGURE 3:**
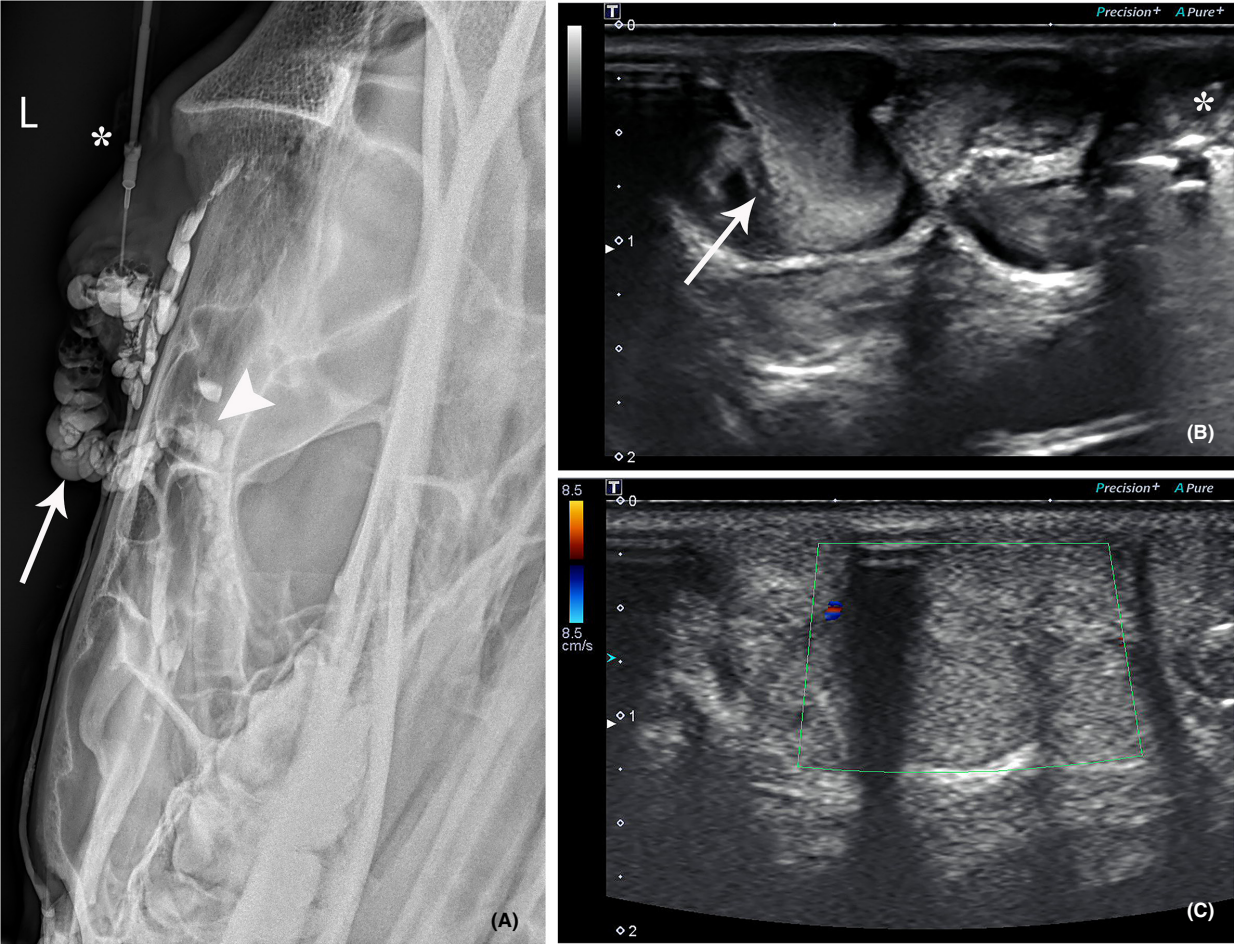
Radiographic and ultrasound images of the superficial orbital venous malformation. (A) Venography revealed the presence of a corkscrew vessel along the ventral aspect of the orbital rim (white arrow), as well as an additional vessel extending ventrally from the region of the medial canthus (white arrowhead). The catheter is visible (asterisk) within the venous malformation. (B) B‐mode ultrasonography performed immediately following the administration of 1% polidocanol with turbulence within the vessel (white arrow). A small amount of gas was also iatrogenically introduced into the vessel at the time of catheterization (asterick). (C) Color flow Doppler ultrasonography image twenty minutes following the administration of 1% polidocanol revealed complete stasis

A radiographic study was performed next using 16 ml of iodinated contrast (Omnipaque 300; GE Healthcare) administered via the catheter. Three orthogonal projections were obtained. Venography confirmed the presence of a corkscrew‐shaped vessel along the ventral aspect of the orbital rim, as well as an additional vessel extending ventrally from the region of the medial canthus (Figure 3A). Based on the volume of contrast used, a volume of 20 ml of sclerosant was deemed sufficient to slightly expand the orbital venous malformation and ensure thorough contact with vascular endothelium.

Following venography, 20 ml of 1% polidocanol solution (Asclera®, Merz Aesthetics) was slowly administered through the catheter. During the administration, a Jaeger eyelid plate and standard scalpel handle were used to apply manual pressure on the medial and lateral aspects of the superficial orbital venous malformation, respectively, to ensure the sclerosant remained within the venous malformation. Ultrasound of the left jugular vein was performed repeatedly to evaluate for visible thrombus formation. Immediately following administration, turbulence was noted within the vessel (Figure 3B). Twenty minutes later, color flow Doppler ultrasonography revealed complete stasis within the venous malformation (Figure 3C), and thus, it was deemed safe to remove the Jaeger eyelid plate and scalpel handle. The patient was hospitalized overnight, and 500 mg flunixin meglumine was given IV 12 h later.

### 2.3 Clinical outcome

**FIGURE 4:**
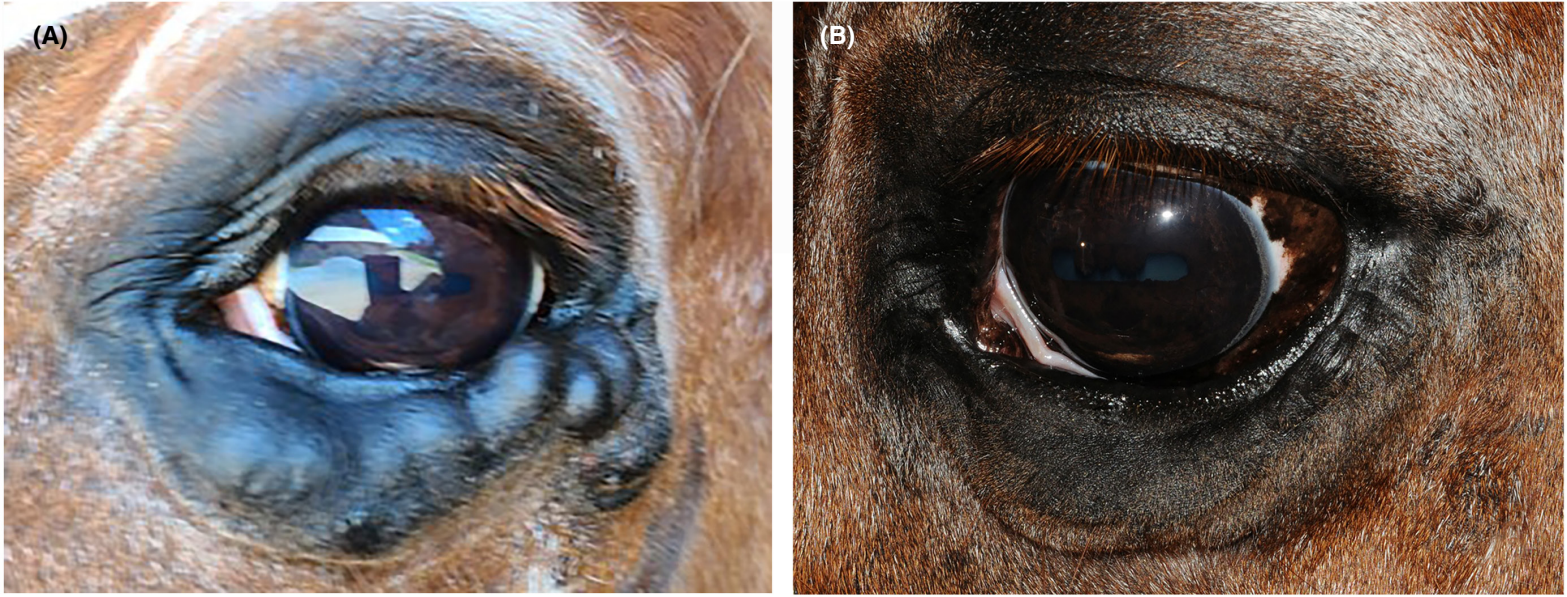
External photographs of the left eye taken (A) 1 month and (B) 4 months following sclerotherapy. Note the (A) gradual improvement in the size of the superficial orbital venous malformation and (B) complete resolution with loose, redundant skin present along the ventral lid margin

No change in the size of the superficial orbital venous malformation was seen the following morning, though it was firm on palpation consistent with thrombus formation. The patient was discharged with OptixCare ophthalmic lubricant (OptixCare Eye Lube; CLC Medica) to be used three times daily for 1 week. One month following sclerotherapy, the venous malformation had reduced in size and the palpebral margin abnormalities and superficial keratitis had improved (Figure 4A). The venous malformation continued to remain firm on palpation. Four months following sclerotherapy, the venous malformation had completely resolved, with loose, redundant skin present along the ventral lid margin (Figure 4B). The palpebral margin abnormalities and superficial keratitis had resolved as well. No adverse side effects were noted at any time during or following sclerotherapy.

## 3 DISCUSSION

This is the first report describing the successful use of 1% polidocanol as monotherapy for a superficial orbital venous malformation in a horse. Based on the patient's history, the venous malformation was suspected to be of congenital origin. Diagnostic imaging was consistent with a venous malformation involving the lateral palpebral and transverse facial veins, and resolution occurred within 4 months following sclerotherapy.

Diagnosis of orbital vascular malformations is dependent upon history, physical examination, and diagnostic imaging. Reported diagnostic imaging modalities include contrast radiography,^2^, ^6^ ultrasonography,^2^, ^4^, ^5^, ^9^, ^11^, ^12^ CT,^3^, ^5^, ^12^, ^13^ and MRI.^4^, ^8^, ^9^, ^11^, ^12^ In this case, contrast radiography and ultrasonography were elected as they could be performed without general anesthesia.

A variety of sclerosants have been reported for treatment of periorbital venous malformations in humans, including ethanol,^14^, ^15^ pinyangmycin (bleomycin A5),^15^ bleomycin (bleomycin A2 and B2),^15^ polidocanol,^14^, ^15^ ethanolamine oleate (EAO),^14^, ^15^ and sodium tetradecyl sulfate (STS).^15^

Ethanol is the most extensively studied and potent sclerosant. It acts by denaturing proteins, thereby damaging the vascular endothelium, leading to thrombus formation and eventual obliteration of the vascular lumen.^9^, ^10^, ^12^ The reported response rate of ethanol for the treatment of vascular malformations of the head and neck is 84%–100%.^15^ This increased potency carries an increased risk of complications, with up to 61% of patients experiencing complications including nerve injury, tissue necrosis, and skin ulceration.^10^ Ethanol administration is quite painful as well, and therefore, patients are typically anesthetized.^9^

Pingyangmycin is the sclerosant of choice in China, due to its high rate of efficacy (>95% response rate) and low risk of complications (average complication rate of 2%). Bleomycin similarly has a low risk of complications (6%), but a more variable efficacy rate (70%–100% response rate).^15^ Pingyangmycin is not commercially available in the United States, and bleomycin was not utilized in this case as it is a chemotherapeutic agent, and therefore, requires special precautions when handling.

Ethanolamine oleate, polidocanol, and STS are detergent‐type sclerosants that lyse endothelial cells and were developed to be safer alternatives to ethanol.^9^, ^10^, ^12^ Minor complications related to treatment occur in 10%–12% of patients treated with detergent sclerosants, compared with up to 50% of patients when absolute ethanol is used.^12^ In one metanalysis, EAO monotherapy had the highest cure rate (59.2%) of all sclerosants, with polidocanol (41.6%) and ethanol (27.1%) having intermediate cure rates, and STS having the lowest cure rate (12%).^14^ When evaluating venous malformations restricted to the cervicofacial region, the overall response rate of EAO ranged from 88%–100%, whereas both polidocanol and STS had a response rate of 100%, though both of these treatment groups were smaller.^15^

Ethanolamine oleate, polidocanol, and STS are all commercially available in the United States. EAO (Ethamolin®, QOL Medical) has been approved by the FDA for treatment of esophageal varices, whereas polidocanol (Asclera®, Merz Aesthetics) and STS (Sotradecol®, Mylan Inc.) are FDA approved for the treatment of varicose veins of the lower extremity.

Ethanolamine oleate, polidocanol, and STS can be administered either as a liquid or a foam. Foamed sclerosants are preferred over liquid sclerosants in certain cases as foamed sclerosants displace blood when administered, leading to increased contact time and use of a lower concentration sclerosant.^16^ However, there is some data indicating that foam sclerosant may be associated with a higher risk of complications, such as visual disturbances and headaches.^17^ In our case, liquid polidocanol was selected due to (1) potential higher risk of complications associated with foamed sclerosant, (2) increased logistical challenges associated with foaming,^18^ and (3) manual occlusion of the proximal and distal ends of the vessel was possible.

Polidocanol was specifically selected in this case because of (1) availability, (2) FDA approval, and (3) reported success in the recent treatment of a nasolacrimal duct cyst^13^ in a canine patient. However, any of the detergent‐type sclerosants would have been reasonable treatment options in this case. Future studies may include the use of different detergent‐type sclerosants.

Complications during or following sclerotherapy were not observed in our study. In humans, there is a low complication rate following sclerotherapy as monotherapy of orbital and periocular venous malformations, with reported complications including swelling, discomfort, skin erythema, skin necrosis, scar formation, orbital compartment syndrome, vision loss, reduced infraorbital nerve sensation,^10^ and most commonly, the need for repeated treatments for resolution.^11^ Adverse effects reflect differences in technique, severity of disease, and sclerosing agents used.^12^

In conclusion, sclerosant monotherapy of a superficial orbital venous malformation using 1% polidocanol in a sedated horse was well tolerated and led to clinical resolution. It is a safe treatment option for superficial orbital venous malformations and should be considered as a minimally invasive treatment option for future cases.

## CONFLICT OF INTEREST

None.
